# Autoantibodies in Rheumatoid Arthritis – Laboratory and Clinical Perspectives

**DOI:** 10.3389/fimmu.2021.685312

**Published:** 2021-05-14

**Authors:** Johan Rönnelid, Carl Turesson, Alf Kastbom

**Affiliations:** ^1^ Department of Immunology, Genetics and Pathology, Uppsala University, Uppsala, Sweden; ^2^ Department of Clinical Immunology and Transfusion Medicine, Uppsala University Hospital, Uppsala, Sweden; ^3^ Rheumatology, Department of Clinical Sciences, Malmö, Lund University, Malmö, Sweden; ^4^ Department of Rheumatology, Skåne University Hospital, Malmö, Sweden; ^5^ Department of Rheumatology, Linköping University Hospital, Linköping, Sweden; ^6^ Department of Biomedical and Clinical Sciences, Linköping University, Linköping, Sweden

**Keywords:** rheumatoid arthritis, rheumatoid factor, ACPA, anti-CCP, diagnosis, prognosis

## Abstract

Measurement of two groups of autoantibodies, rheumatoid factor (RF) and anti-citrullinated protein/peptide antibodies (ACPA) have gained increasing significance in the diagnosis and classification of rheumatoid arthritis (RA) over the last 65 years. Despite this rising importance of autoimmune serology in RA, there is a palpable lack of harmonization between different commercial RF and ACPA tests. While a minimal diagnostic specificity has been defined for RF tests, which almost always are related to an international reference preparation, neither of this applies to ACPA. Especially assays with low diagnostic specificity are associated with very low positive predictive values or post-test probabilities in real world settings. In this review we focus on issues of practical bearing for the clinical physician diagnosing patients who potentially have RA, or treating patients diagnosed with RA. We advocate that all clinically used assays for RF and ACPA should be aligned to a common diagnostic specificity of 98-99% compared to healthy controls. This high and rather narrow interval corresponds to the diagnostic specificity seen for many commercial ACPA tests, and represents a specificity that is higher than what is customary for most RF assays. Data on antibody occurrence harmonized in this way should be accompanied by test result-specific likelihood ratios for the target diagnosis RA on an ordinal or interval scale, which will provide the clinical physician with more granular and richer information than merely relating numerical values to a single cut-off point. As many physicians today are used to evaluate autoantibodies as positive or negative on a nominal scale, the introduction of test result-specific likelihood ratios will require a change in clinical mindset. We also discuss the use of autoantibodies to prognosticate future arthritis development in at-risk patients as well as predict severe disease course and outcome of pharmacological treatment.

## Introduction

Autoantibody measurements have been long-term companions to physicians involved in the management of rheumatoid arthritis (RA) patients, with increasing importance during the last decades. As guidelines and criteria nowadays tend to put increasing emphasis on autoantibody analyses, and as the field is highly dynamic, it becomes even more important for physicians to be aware of pitfalls and advantages of such testing. Thus, we aimed to overview the current ‘serological landscape’ in RA, from both laboratory and clinical perspectives

## Laboratory Perspectives

### Autoantibodies in Diagnostic and Classification Criteria for Rheumatoid Arthritis

Already in the diagnostic criteria for RA proposed in 1956 (1), a positive sheep cell agglutination test or a positive streptococcal agglutination test (2) was included among the criteria for definite or probable RA. A definite RA diagnosis required five out of 11 criteria, and thus immune serology could constitute up to 20% of the criteria needed. The 1956 criteria (1) did not define what laboratory finding should constitute a positive reaction for RF, but the 1958 revised criteria (3), stated that any method to measure RF could be employed if “positive in not over 5% of normal controls” in two different laboratories, alternatively by a positive streptococcal agglutination test (3). In the 1987 American Rheumatism Association revised criteria for the classification of RA (4), RF constituted one of 7 classification criteria, and as RA was defined by the presence of four or more criteria, autoimmune serology could constitute up to 25% of the criteria needed for classification as RA. The definition of a positive RF reaction was slightly modified to “abnormal amounts of serum rheumatoid factor by any method for which the result has been positive in <5% of normal control subjects”. Thus, a specificity more than, but not including, 95% was employed.

Major discoveries prompted the development of new criteria. The advent of biological therapies had dramatically improved the prognosis for RA patients (5). A new understanding emerged concerning the “window of opportunity” within the first weeks after appearance of RA symptoms, when active treatment with disease-modifying anti-rheumatic drugs (DMARDs) should be commenced, and that delayed start of RA treatment had long-term negative effects (6). Collectively, these circumstances led to criticism of the 1987 classification criteria for lacking sensitivity in early RA. The discovery of anti-citrullinated protein/peptide antibodies (ACPA) also changed the scene (7, 8). The 2010 European League against Rheumatism (EULAR)/American College of Rheumatology (ACR) classification criteria for RA therefore focus on discriminating between high and low risk for persistent or erosive disease among patients presenting with recent onset of synovitis (9). In the 2010 criteria, both RF and ACPA are included, and a score of 6 or greater out of 10 possible classify as RA. Intriguingly, the 2010 classification criteria do not convey any traceable information about how to define the occurrence of RF or ACPA, and negative values are referred to as “less than or equal to the upper limit of normal (UNL) for the laboratory and assay” (9). Low positive values were defined as between 1-3 times the UNL, and high positive values > 3 times the UNL for the laboratory and assay. Low levels of RF or ACPA yield a score of 2, and high levels yield a score of 3. Qualitative RF responses yield a score of 2 (9).

Consequently, autoantibodies may now account for up to 50% of the scores needed to classify as definite RA, meaning that the impact of autoimmune serology has gradually increased since the first diagnostic criteria in 1956.

At the time of publication of the 1956, 1958 and 1987 criteria, RF was commonly performed with manual techniques locally adopted in individual hospital laboratories. This situation has changed dramatically, and today most laboratories use commercial assay systems comprising ready-made assay kits or fully automated assay systems provided by industrial manufacturers. Since 2017, the In Vitro Diagnostic Medical Device Regulation (IVD-R) describes the regulatory basis for placing new *in vitro* tests on the market in the European Union (10). The IVD-R states that the manufacturing company is responsible for performing clinical validation including determination of diagnostic sensitivity and diagnostic specificity. The reference ranges suggested by the manufacturer are thereafter often accepted after being verified in smaller groups of subjects in the individual clinical laboratories utilizing the corresponding reagents. In practice, reference ranges for RF are commonly defined according to the 1987 classification criteria (4), whereas reference ranges for ACPA are decided at the discretion of the individual companies producing ACPA assay reagents.

In the 1956 criteria, high concentration of lupus erythematosus (LE) cells in blood constituted an exclusion criterion (1, 11). This exclusion criterion remained in the 1958 revision, but was commented as LE cells had been observed in patients with typical clinical features of RA (3). However, anti-nuclear antibodies detected with immune fluorescence (IF-ANA), i.e. the clinical laboratory successor of the LE cell test (12), is quite common among RA patients. In a Swedish study of 105 patients with established RA, IF-ANA was detected in 38% (13). In another study, a positive reaction was found in 20% of 385 patients with early RA classified according to the 1987 criteria (14). In both studies, the diagnostic specificity for IF-ANA was 95% when compared with healthy controls, as suggested by the international recommendations (15). IF-ANA is thus common among RA patients and consequently, this exclusion criterion was omitted in the 1987 and 2010 RA classification criteria (4, 9).

### Laboratory Techniques Used to Measure RF

RF was originally described using hemagglutination of sensitized sheep red blood cells in an agglutination test (16, 17), with reagents prepared in-house by each laboratory. Later more stable tests appeared based on the agglutination of latex-containing particles of uniform size instead of sheep red blood cells (18). Large scale automation was made possible with the development of nephelometric (19, 20) and turbidimetric (21) techniques. Until then, all methods had been isotype-nonspecific, although they all, due to assay format, mainly detected IgM RF. With the development of isotype-specific ELISAs (22) and other enzyme immunoassays, this hurdle was overcome. There are also examples of commercial addressable laser bead immunoassays (ALBIA) for the measurement of RF (23).

The report for the October 2020 distribution from the British National External Quality Assurance Scheme (UK NEQAS) contained 312 responses for RF (308 correctly reported positive). RF had – in different laboratories - been analyzed with four latex agglutination methods, although no laboratory reported measurement with the original hemagglutination technique. Other techniques reported were one chemiluminescence method, 8 enzyme immunoassays, 12 turbidimetry methods, two nephelometry assays, and one addressable laser bead immunoassay ALBIA. Only one laboratory reported using an in-house ELISA to measure RF, whereas all other laboratories stating details used commercial tests.

### Clinically Used Assays for ACPA Determination

A number of different commercially available ACPA tests have been developed, detecting antibodies that target different citrullinated proteins and peptides. The first assay marketed in 2000 used a defined peptide from filaggrin, the citrullinated autoantigen in anti-keratin antibodies (24), and the first protein to be used as a citrullinated autoantigen in RA studies (7, 8). The public peptide sequence was made cyclic by oxidative folding between thiol groups in two cysteine residues to allow more efficient recognition of the citrullinated epitopes by ACPA. Consequently, the antigen was denoted cyclic citrullinated peptide (CCP) (25). By screening around 12 million peptides from synthetic libraries with RA sera, a new set of peptide(s) was incorporated into assays denoted cyclic citrullinated peptide version 2 (CCP2) (26). A great number of studies have shown that anti-CCP2 defines RA patients with poor prognosis, both concerning inflammation and radiographic joint damage (27, 28). Comparative studies clearly showed that anti-CCP2 had higher diagnostic sensitivity at equal specificity, and also defined more patients with poor radiological prognosis, compared to anti-CCP1, which was the name now given to the original anti-CCP test (29). The proprietary CCP2 has been licensed to many diagnostic companies which produce anti-CCP2 tests, and one company developed their own cyclized peptide denoted CCP3 which also has good diagnostic qualities (30, 31). A German company developed a test based on mutated and citrullinated vimentin, denoted anti-MCV (32). Although anti-MCV could detect patients with poor radiological prognosis also among anti-CCP2-negative patients (33), and high levels of anti-MCV have been particularly associated with severe extra-articular manifestations of RA (34), a number of studies have raised issues concerning the diagnostic performance of anti-MCV, especially in the high specificity part of the receiver operator characteristics (ROC) curve (35, 36). A commercial ELISA based on recombinant citrullinated rat filaggrin was also developed (37, 38), and an Italian company has established an assay based on a viral citrullinated peptide (VCP2) from Epstein-Barr virus-encoded protein (39).

In Europe, the anti-CCP2 test provided by different companies and in different assay formats is the dominating test. Although the absolute majority of commercial ACPA tests measure IgG ACPA, some companies have developed commercial IgA and IgM ACPA tests primarily for research purposes (40, 41), and one company developed a variant ACPA test denoted anti-CCP3.1 with mixed anti-IgG/anti-IgA conjugate (31, 42).

A large number of ACPA fine specificities have been described, also appearing in the anti-CCP2 negative RA subset (43). However, no such fine specificities have gained widespread clinical use.

The October 2020 quality assessment distribution from UK NEQAS contained 407 responses from individual laboratories for ACPA, with 406 correctly reported positive. ACPA had been analyzed with 6 different chemiluminescence methods, 11 enzyme immunoassays and one luminex-based assay. All laboratories used commercial ACPA tests.

### Non-Criteria Autoantibodies in RA

Besides RF and ACPA, other groups of antibodies have been implicated as diagnostic and/or prognostic biomarkers in RA. ACPA belong to a group of antibodies against post-translationally modified (PTM) proteins/peptides. Antibodies against carbamylated or homocitrulline-containing proteins (anti-CarP) were originally detected in 45% of RA patients and reported as distinct from ACPA based on inhibition studies (44). Anti-CarP predicts poor radiological outcome in early arthritis patients (45). A meta-analysis suggested high specificity but relatively low sensitivity for anti-CarP (46). Together with antibodies against acetylated residues, ACPA and anti-CarP are collectively termed anti-modified peptide antibodies, or AMPA (47). The original studies claiming non-cross reactivity used rather complex ELISAs with carbamylated fibrinogen or carbamylated fetal calf serum as antigens, and polyclonal patient sera. Later studies, which used small peptides with different individual PTMs (48) and/or monoclonal AMPA from RA patients (49, 50) have shown extensive cross-reactivity, especially between ACPA and anti-CarP. Antibodies against peptidyl arginine deiminase-4 (PAD-4), an enzyme responsible for citrullination, was originally detected in 36-42% or RA patients with high specificity (51), and gained interest as anti-PAD-4 could inhibit citrullination of fibrinogen (52). A meta-analysis has suggested rather low diagnostic sensitivity but high specificity for anti-PAD-4 (53). Antibodies against glucose-6-phosphate isomerase (anti-GPI), distinctively pathogenic in the K/BxN T cell receptor transgenic mouse arthritis model, were first described in 64% of RA patients but not in controls (54). Later studies, however, showed anti-GPI also in other arthritides and systemic rheumatic diseases (55, 56). Type II collagen (CII), the most abundant antigen in hyaline cartilage, is an autoantigen in animal arthritis models, and anti-CII in RA was first described almost 50 years ago (57). More recent studies have described high levels of functionally active cytokine-inducing anti-CII in a limited group (5-10%) of newly diagnosed RA patients. As anti-CII levels drop during the first year, so does the anti-CII induced inflammation. Anti-CII might therefore be a marker for an acute onset RA subgroup with good prognosis (58, 59). Heterogeneous nuclear ribonucleoprotein A2, or RA33 is a target for autoantibodies in about one third of RA patients, but also in systemic lupus erythematosus (SLE) and mixed connective tissue disease patients with antibodies against DNA and the Sm/RNP complex (60). A recent meta-analysis reported pooled sensitivity and specificity values of 31.8% and 90.1%, respectively (61). Antibodies against products of lipid degradation, malondialdehyde (MDA) and malondialdehyde-acetaldehyde (MAA) are increased in RA and show some association to RF and ACPA (62). The levels increase before diagnosis of RA, albeit at a later stage than RF and ACPA (63). Antibodies against the immunoglobulin binding stress protein BiP have been found in sera both from RA patients and asymptomatic subjects subsequently developing RA (64); but a recent meta-analysis showed only moderate diagnostic sensitivity (65). Antibodies against calpastatin were described more than 25 years ago in 57% of investigated RA patients (66). Anti-agalactosylated IgG autoantibodies have been described in 83% of RA patients, but comparison with disease controls showed lower specificity than for anti-CCP (67).

None of these non-criteria autoantibodies have obtained widespread use, although anti-CarP has gained significant interest in a scientific context. Henceforth, we will focus on the clinical use of RF and ACPA.

### International Reference Preparations for RF and ACPA

The first World Health Organization (WHO) RF standard was produced by pooling RA sera collected in 1963. In 1964 the pool was divided into three batches, where the first formed the international reference serum denoted W1066 (68). The second batch formed the 1^st^ British standard denoted 64/002 (69). As they are from the same source, W1066 and 64/002 are interchangeable. Eleven laboratories from seven countries participated in the collaborative study where all participants were asked to use sheep cell agglutination, and no isotype specific techniques were in use at that time. The 1^st^ WHO standard W1066 was described in 1970 (70) and has been available *via* the National Institute for Biological Standards and Control (NIBSC) in United Kingdom (www.nibsc.org). The majority of commercial tests for RF are standardized against W1066, and the unitage is consequently given as international units (IU)/ml.

The first reference preparation for ACPA prepared from defibrinated plasma from one strongly ACPA-positive RA patient diluted in a pool of ACPA negative serum samples was described in 2012 (71). Twelve commercial methods, the majority based on the CCP2 antigen were investigated in parallel. Except the anti-CCP3.1 test detecting both IgG and IgA ACPA, the other 11 assays only detected IgG ACPA. When dilutions of the reference sample was used as a calibrator in the different assays, the mean coefficient of variation was reduced from 76.4% to 27.9% for samples with medium/high ACPA levels (71). The reference preparation is available from the Antibody Standardization Committee (ASC), a subcommittee of the International Union of Immunological Societies (IUIS) quality assessment and standardization committee (72). Although it belongs to the reference preparations colloquially called the “CDC reagents”, the IUIS/ASC reference preparation is today distributed *via* the Plasma Services Group (www.plasmaservicesgroup.com). To our knowledge, no commercial ACPA test has so far been standardized against this preparation.

A tentative new candidate material named 18/204 has been investigated in a collaborative study led by NIBSC, with the aim to produce a new WHO standard for RF and ACPA. The candidate material was also evaluated by the European Consensus Finding Study Group on Autoantibodies (ECFSG) in 2019-2020. The complexity of the results from the international collaborative study has raised some unexpected questions, and the approach for using 18/204 as an RF/ACPA standard or reference reagent is still under consideration (Lucy Studholme, personal communication).

### Standardization of Autoantibody Analyses in the Clinical Laboratory

In Sweden, most if not all laboratories performing autoantibody analyses are accredited according to EN/ISO 15189:2012 standard (73). This document is general, and does not fulfill all needs concerning instructions for immunological laboratories. A consensus document was recently published to fill these needs and to create a framework for accreditation purposes (74), including internal controls and external quality assessment schemes. Internal controls (both positive and negative) are individual samples included in all performed analyses in parallel to patient samples. One positive sample should preferably have a value close to the assay cut-off, where stability should be secured (74). Acceptable variation, usually given as % coefficient of variation around the mean, are predefined and repeated deviations outside that range should lead to report to the laboratory manager for further actions. Internal control samples provided with assay kits can change with new lots of reagents in ways unpredictable for the clinical laboratories. Consequently, it is of great value to have enough of own kit-independent internal controls to allow continuous analysis over time covering changes between different reagent lots. It is also optimal to have internal controls from single patients (obtained from plasmapheresis), as variations between different batches of assay kits tend to be more evident with single donor controls than with pooled controls (75). However, such large quantities of single donor sera are seldom available, and laboratories often use pools of anonymized patient samples as internal controls.

**Figure 1:**
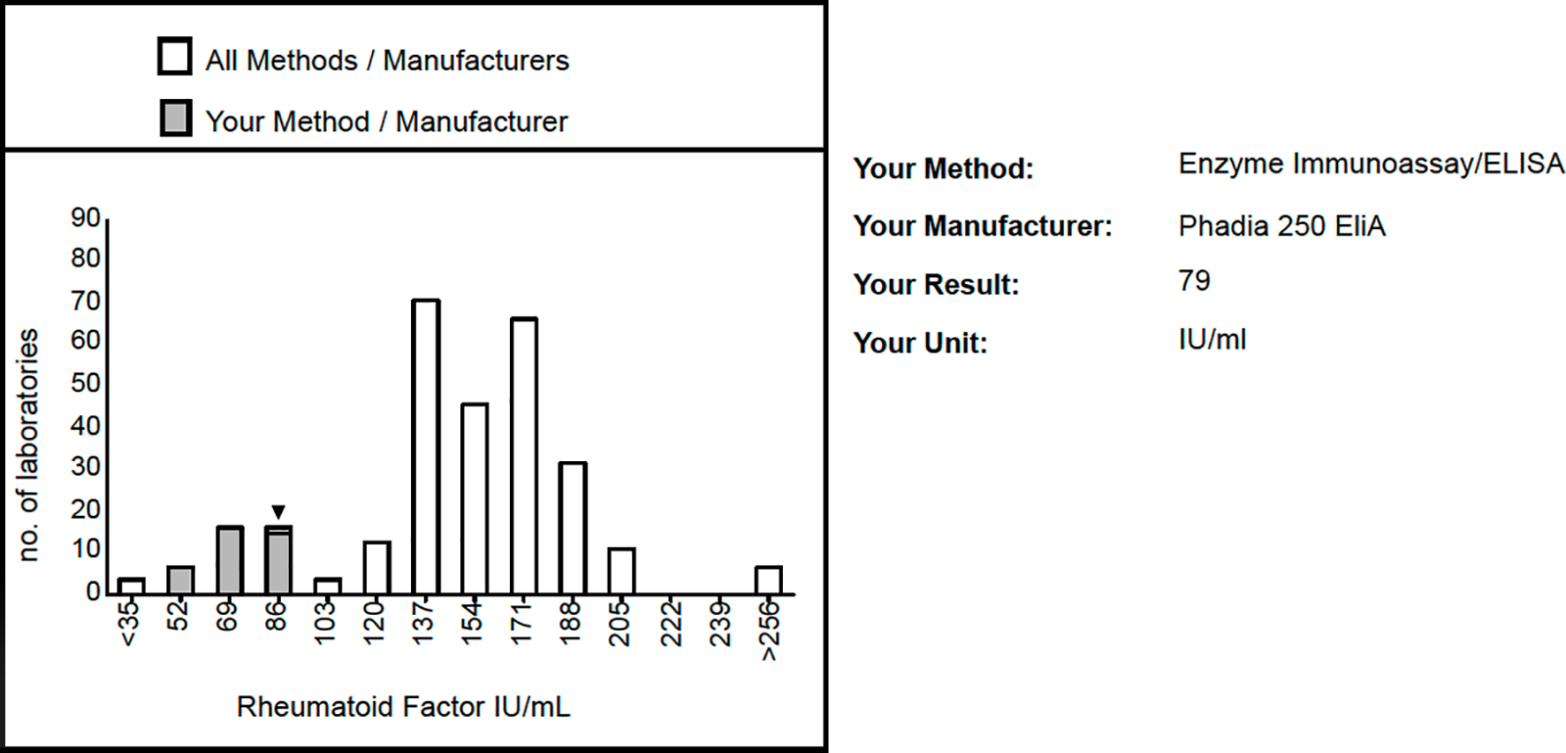
Excerpt from the response from the British External Quality Assessment provider UK NEQAS to one individual laboratory on the October 2020 distribution of rheumatoid factor. Responses had been submitted from 312 laboratories, out of which 308 were correctly positive and four incorrectly reported as negative. The histogram bars show the quantitative distribution for all participating labs, with the distribution of labs using the same commercial assay as this individual laboratory in grey. The figure is published with the permission of Dina Patel, UK NEQAS.

External quality assessment (or proficiency testing) programs are conducted by independent bodies who dispatch samples, often 4-6 times/year to participating laboratories. The laboratories perform the prescribed analyses and return the results to the external quality assessment provider who compile the data and thereafter return back the individual assessments. **Figure 1** shows an excerpt from such a report for RF from UK NEQAS.

#### Variability Between Methods to Measure RF and ACPA

There is often an obvious discrepancy between quantitative results from RF measurements performed with different methods. Already one of the first studies on RF measured with nephelometry noted only a modest correlation between agglutination test titers and nephelometry (r=0.46) after excluding seronegative patients (19). Comparisons between nephelometry and turbidometry have also showed significant differences, especially in the low positive range (76), and even different IgM RF immunoassays have shown clear discrepancies depending on whether the target antigen source was human or rabbit IgG (77).

There is also a considerable variation between different ACPA tests, although they are methodologically more similar and all use citrullinated peptides or proteins bound to solid phases in immunoassays. In a comparison between six different ACPA assays targeting citrullinated filaggrin, MCV, CCP2 (three assays) and CCP3, diagnostic sensitivity ranged between 69.6% and 77.5% and diagnostic specificity between 87.8 and 96.4%. However, the areas under the ROC curves (AUC) were similar, and there was a good correlation between quantitative values for the three anti-CCP2 tests, with r values between 0.90 and 0.95 (37). In an Italian study where 11 different commercial ACPA assays were compared investigating 100 RA patients and 202 healthy and disease controls, the AUC were largest for assays using CCP2 or anti-CCP3 as antigens, but lower when other citrullinated antigens (filaggrin, vimentin, IgG, Epstein Barr virus) were used. ROC curve analyses suggested widely differing sensitivities and specificities, but when all cutoffs were adjusted to the same diagnostic specificity (98.5%), the assays with lowest AUC also showed the lowest diagnostic sensitivities; highest sensitivities were found for the anti-CCP assays. Again, there was an almost perfect agreement between assays using CCP2 and CCP3 antigens. The authors concluded that the most important variable for assay accuracy is the source of antigen and that other variations in kit preparation are secondary (38). A third study from Belgium recently investigated 594 consecutive patients seeing a rheumatologist in a real world setting, and being tested for RF and ACPA for the first time. Diagnoses were reviewed by the consulting rheumatologist, and reviewed again after one year of follow-up. The authors found large variations in sensitivity and specificity between assays, notably mainly for RF (78).

In all these studies, numerical ACPA values differed widely between assays, as there is no commonly used international standard for ACPA. Two studies have therefore compared the ratios between the values obtained for the IUIS/ASC ACPA standard and the cut-offs suggested by the manufacturers for different commercial assays. In the study describing the IUIS/ASC ACPA standard, this was done for 12 commercial methods, with a ratio between 5.6 and 28.5 (71). As this ratio differed more than five times between the extremes, it reflects a more than five time difference in recommended cut-offs, which are often implemented by clinical laboratories and which in the 2010 EULAR/ACR classification criteria are called “upper limit of normal (ULN) for the laboratory and assay” (9). Expressed differently, it means that the same sample might either get zero points (negative), 2 points (between 1-3 ULN) or 3 points (≥3 ULN) in the 2010 criteria, depending on what assay was used (9). The Belgian study referred to above also performed such calculations, and found lower degree of variability with ACPA ratios between 11.2 to 22.3, i.e. a twofold difference. When they on the other hand calculated ratios between the international RF standard W1066 and individual RF assay cutoffs, the ratios differed between 0.6 and 9.3, a 15-fold difference. Consequently, there was a large variation in sensitivity and specificity between assays, especially for RF. The authors concluded that, depending on assay used, patients might or might not be classified as having RA (78).

### How Cut-Offs for RF and ACPA Are Determined

When the diagnostic performance of different autoantibody assay systems is compared, it is generally recognized to use all assays in parallel to investigate the same groups of patients and controls, primarily including disease controls with a clinical phenotype mimicking the target diagnosis. However, results are often presented with varying values both for diagnostic sensitivity and diagnostic specificity for the individual tests (78, 79), often because the authors have used the manufacturer-suggested cut-offs. As discussed earlier, when cut-off points from different assays measuring the same autoantibody are related to each other, they differ up to five times for ACPA and up to 15 times for RF (71, 78). Without knowledge about the actual shapes of the corresponding ROC curves in the important upper left part, and about cut-offs corresponding to individual points on the ROC curves, such data are very difficult, if not impossible to interpret correctly.

There is also a general trend that the cut-off values for RF tests are set at a lower specificity than for ACPA (78). This is probably at least partly due to the 1987 ACR classification criteria stating a specificity of > 95% (4) whereas the first ACPA studies evaluating ACPA levels with ELISA usually used a cutoff level corresponding to 98%-99% specificity (25, 80). Due to a rather low specificity, the positive predictive value (PPV) for RF can be very low in health care settings where RA is uncommon. In a US study performed in a teaching hospital on 563 analyses, the PPV for RA was 24% (81). In a recent Danish real-world retrospective population-based registry study on patients where ACPA and RF were ordered in 60300 patients between 2007 and 2016, 5% of the investigated patients developed RA. The PPV was higher for ACPA (30%) than for IgM RF (12%) when the cutoffs suggested by the assay manufacturers were used (82). Higher PPVs for ACPA (43%) than for IgM RF (14%) remained also when a cutoff corresponding to three times UNL was used (83). As pointed out by the authors of the American study (81), the selection of patients among whom an RF test is performed probably matters as much or more than the characteristics of the individual RF assays.

**Figure 2:**
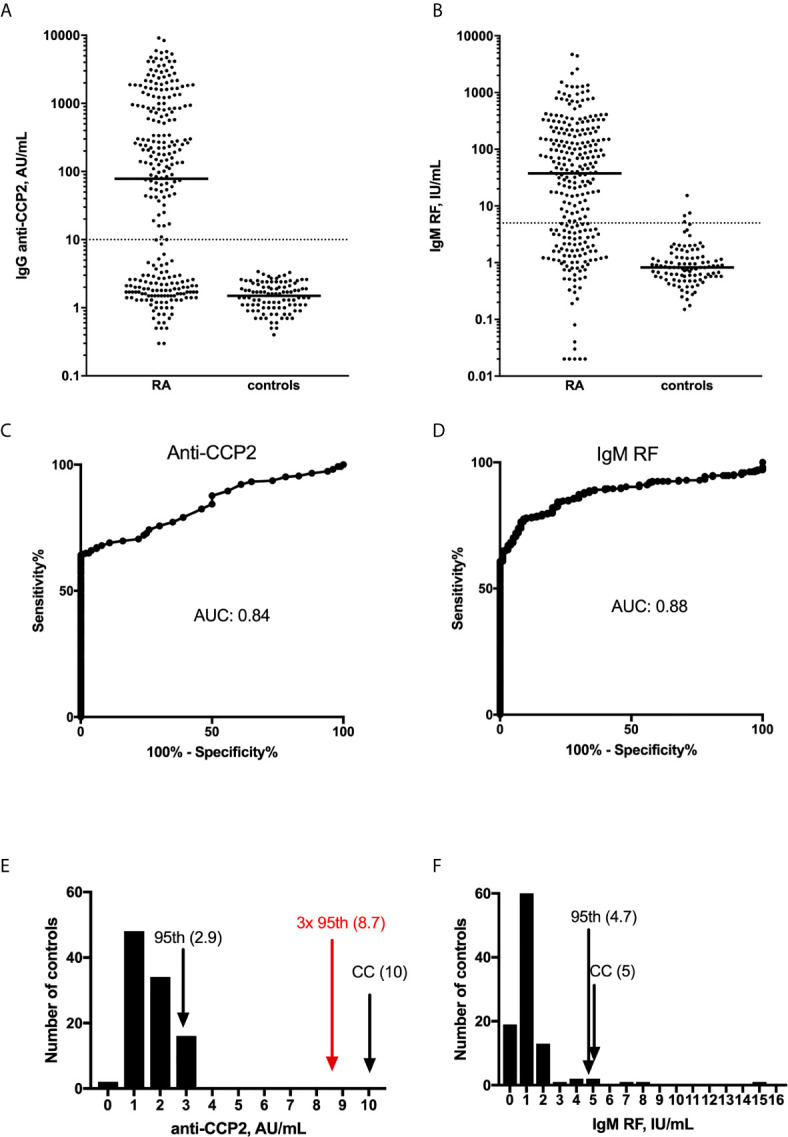
Distribution of **(A, C, E)** anti-CCP2 and **(B, D, F)** IgM RF among 268 RA patients and 100 healthy blood donors from Sweden. In **(A, C)**. dot blots are shown with the medians depicted as horizontal solid lines. The dotted horizontal lines depict the cut-off points for clearly positive responses, as suggested by the manufacturer. In **(B, D)**, the corresponding Receiver Operator Characteristics (ROC) curves are shown; including information about Area Under the Curve (AUC). In **(E, F)** the distribution of the 100 controls is depicted for anti-CCP2 and IgM RF, with vertical arrows depicting the 95th percentile among the 100 controls (95th), the company-suggested cutoffs (CC), and in **(E)** the value three times higher than the 95th percentile (3x 95th, in red). Figures within parentheses show the corresponding measurement values.

It is easier to intuitively recognize a plausible cutoff for ACPA than for RF. In **Figures 2A, B** we show IgG anti-CCP2 and IgM RF values both measured with the Phadia Elia system in a cohort of 268 previously described Swedish RA patients (28, 58), together with 100 healthy blood donors. All samples with levels above the measurement range were further diluted and re-assayed to obtain quantitative information for all individuals. The distribution of anti-CCP2 (**Figure 2A**) for the patients is clearly more bimodal and with a thinner waist than for IgM RF (**Figure 2B**), in agreement with studies arguing that ACPA positive and ACPA negative RA are separate disease entities with different genetic and environmental risk factors (84). The corresponding ROC curves are depicted in **Figures 2C, D** whereas the distributions among the healthy controls are shown in **Figures 2E, F**. The 95^th^ percentile for IgM RF amounts to 4.7 international units (IU)/mL (**Figure 2F**), which is in agreement with the 5 IU/mL cutoff suggested by the manufacturer which, in turn, is in agreement with the 1987 classification criteria stating >95% diagnostic specificity (4). The 95^th^ percentile for anti-CCP2 corresponds to 2.9 arbitrary units (AU)/mL, which is much lower than the 10 AU/ml cut-off suggested for clearly positive results by the manufacturer (with a suggested equivocal range between 7-10 AU/mL). In fact, if the same specificity level would apply for anti-CCP2 as for IgM RF to determine cut-off or UNL, 3 times UNL, i.e. the level resulting in three points in the most recent classification criteria (9) would be lower than the cutoff for a clearly positive reaction currently suggested by the manufacturer (red arrow, **Figure 2E**). The figure exemplifies the trend of generally higher diagnostic specificity for ACPA tests than for RF assays in the current practice (78).

### Alternative Approaches to Report Results for RF and ACPA to the Physicians

**Figure 3:**
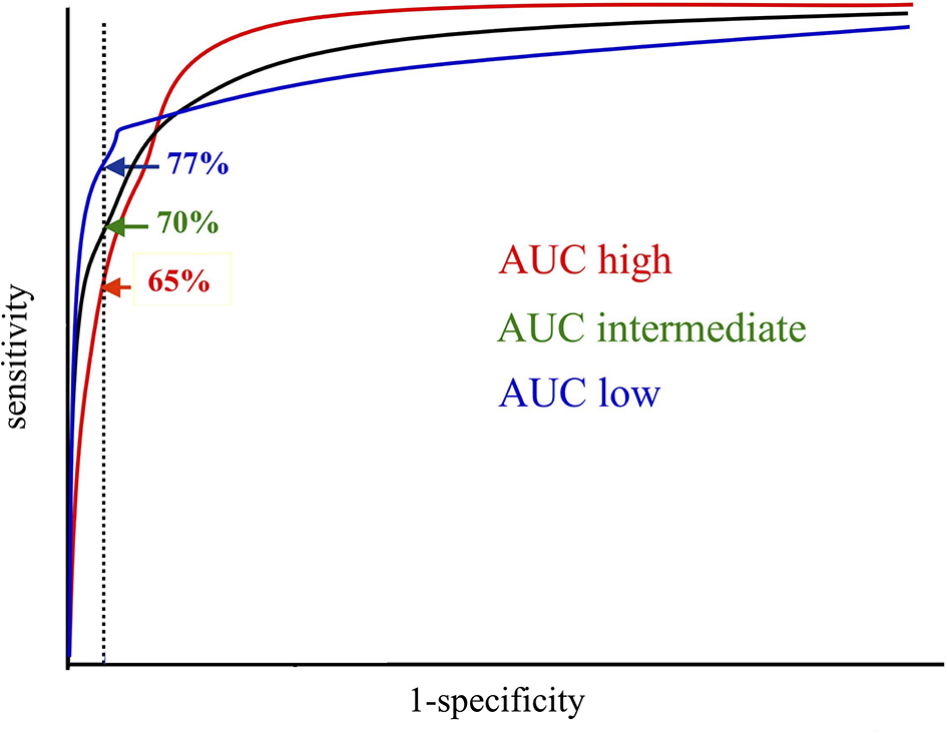
When diagnostic sensitivities are compared between different tests, they should be aligned to the same diagnostic specificity, preferably in the high specificity range. In this schematic figure, the Area Under the Curve (AUC) is highest for the red and lowest for the blue Receiver Operator Characteristics (ROC) curve. However, at the predefined diagnostic specificity (vertical dotted line) the blue ROC curve represents the test with the highest sensitivity, which should be preferred when laboratory results are reported in relation to one single cutoff. The original picture was obtained from Allan Wiik, Copenhagen, and published in modified form with his permission.

We suggest that assays for the investigation of RF and ACPA should have a standardized specificity range, and that this specificity range should be rather high and rather narrow, between 98-99%. We also propose that this range should be the same for RF and ACPA to enhance comparability between the two autoantibody tests and to increase the positive predictive values of RF tests which today are very low in real-world settings (81–83). Such a defined range with an upper limit is more specific than, but not in conflict with, the 1987 ACR classification criteria which by stating >95% specificity formally do not rule out higher cut-off settings (4). In such a cut-off focused approach, the AUC of the ROC curve is of limited importance, especially in the right low-specificity range, see **Figure 3**. In this schematic figure the ROC curve with the largest AUC has the lowest sensitivity at the pre-defined high specificity, whereas the ROC curve with smallest AUC has the highest sensitivity at the pre-defined specificity level, given the ROC curve shape with close alignment with the y axis in the high specificity range (**Figure 3**).

Establishment of cut-off levels in the high specificity range demands large control populations. To establish a 99^th^ percentile cutoff with a 95% confidence interval, at least 678 controls have to be investigated (85). The establishment of these cut-off values has to be within the responsibility for the validation performed by the manufacturing companies (10), as the much smaller verification performed at each laboratory before introducing a new laboratory assay can never encompass such workload and costs. The size and complexity of such an undertaking, together with the need for carefully characterized patient populations, argues for a joint effort between diagnostic industry and the main professional bodies within rheumatology, e.g. EULAR and ACR.

We suggest that in this situation the cut-offs will remain related to healthy controls and not to disease controls, as described in the 1987 classification criteria (4) and never changed since then. Disease controls encompassing patients with differential diagnoses to the target diagnosis and consulting the clinician in the same clinical setting as the target diagnosis patients are better comparators to define which levels are clinically important in the real life situation (86). However, it is very difficult if not impossible to define or standardize RF levels in “disease controls”, even when defined by discrete diagnoses, and thereby the objective of cut-off level harmonization will not be reached. To paraphrase Leo Tolstoy, who as a novelist had the artistic freedom to simplify: healthy controls are all alike; every disease control is diseased in its own way (87).

Even with aligned specificities, this analysis result would not respond to the question asked by the rheumatologist at the patient’s bedside or in the outpatient clinic. Although sensitivity and specificity tell you what fraction of patients with RA or control individuals will have RF or ACPA respectively, the clinician frequently seeks the answer to the following question: what is the probability that the patient in front of me has RA given that I get a positive (or negative) result of the RF or ACPA tests? Or even more informative: what is the probability of disease given that the level of RF or ACPA is within a certain range? These probabilities can be calculated given knowledge on sensitivity, specificity and the risk for RA in an individual patient before autoantibody testing given the individual patient’s unique set of risk factors, or alternately at the population level, the prevalence of RA in the investigated group of patients.

It is based on Bayesian statistics based on a theorem described by the reverend Thomas Bayes in the 18^th^ century (88) and which subsequently was incorporated into clinical decision making (89–91).

The likelihood ratio (LR; the ratio between the likelihood of a test result in patients and the likelihood of the corresponding result in controls) is not depending on prevalence, but on the patient and control groups used. By knowing the pre-test probability or prevalence and the positive LR, the post-test probability or positive predictive value can be calculated (90). In a meta-analysis of 37 studies on anti-CCP and 50 studies on RF, the pooled positive LR for anti-CCP was 12.46, and for IgM RF the corresponding figure was 4.86 (92). These figures should be understood in the context that positive LRs above 10 usually indicate large and often clinically important increase in likelihood of disease, whereas LRs between 2-5 indicate small increase in likelihood of disease (90). LRs were based on the cut-offs used in the included studies, and thus were calculated only for positive and negative reactions. More granular and richer information can however be obtained if LR are determined for different quantitative intervals of RF and ACPA, which can help the clinical rheumatologist to interpret the results in a more nuanced way. In a study from 2009, Bossuyt et al. calculated interval-specific positive LRs for anti-CCP2 (3 intervals) and RF measured with nephelometry (4 intervals). The positive LR for the highest interval was 27.7 for anti-CCP2, and 4.8 for RF; the latter roughly comparable to the positive LR for the middle interval for anti-CCP2 (93). The reasoning is further developed in (94) and in relation to individual commercial RF and ACPA tests in (78), where LRs were stratified both in relation to the company-suggested cut-offs and with all cut-offs aligned to 98.5% specificity.

Cut-off values and LRs are related. Although different commercial assays showed widely differing LRs at the cut-offs recommended by the manufacturers, the LRs became obviously more similar when the cut-offs for the different tests were aligned to the same diagnostic specificity, as has been shown both for RF (78) and ACPA (31, 78) assays.

A position paper arguing for a similar approach in the reporting of anti-proteinase 3 and anti-myeloperoxidase levels as interval-specific likelihood ratios in patients with suspected ANCA-associated vasculitides was recently published (95).

We believe that a combination of reporting ACPA and RF results with cutoffs aligned to a common high specificity range, together with reporting of interval-specific likelihood ratios will both increase the repeatability and granularity of data and thus help clinicians to better interpretation of the clinical significance of laboratory results.

We are aware that this will demand a change in clinical mindset away from viewing autoantibody occurrence as dichotomous information, to instead be interpreted on an ordinal or interval scale. This means moving from treating occurrence of autoantibodies in a binary way as when reviewing an x-ray image for fracture or no fracture, and rather interpret autoantibody data as when a clinician evaluates discrete blood pressure levels being associated with different risks for cardiovascular disease. A practical problem is that the same groups of patients and controls should be evaluated with all tests when comparing LRs between different assays.

## Clinical Perspectives

### Autoantibodies in Patients at Increased Risk of RA

Autoantibody patterns prior to RA onset are being increasingly investigated regarding their prognostic value in clinical practice. Although the occurrence of RA-related autoantibodies prior to symptom onset, which has been described in several previous studies using large biobanks from population surveys or blood donors (96–98) is very interesting from a pathophysiological point of view, physicians mostly encounter patients seeking care due to musculoskeletal pain. Therefore, this overview focuses on subjects with symptoms instead of asymptomatic at-risk populations such as symptom-free first-degree relatives.

### Autoantibodies Before RA Diagnosis

In many countries, autoantibody status is an important determinant leading to referral of symptomatic patients from primary care to rheumatology clinics. Hence, prospective studies constituting of symptomatic patients regardless of autoantibody status are sparse. However, the clinical practice in the Netherlands, where referral of patients is predominately based on symptoms and not autoantibody results, enables such a study design. Thus, ten Brink and colleagues studied 241 arthritis-free yet symptomatic patients (99). Despite a rather strict symptom definition (small joint arthralgia, duration <12 months, and rheumatologist’s suspicion of progression to arthritis), 2-year progression to arthritis was only 10% among patients negative for anti-CCP2, RF, and anti-CarP. Increased arthritis risk estimates were apparent for all 3 autoantibody classes, but anti-CCP2 conferred the highest risk [hazard ratio (HR) 8.5], and was the only autoantibody remaining significant in multivariable analysis. Anti-CarP analysis in addition to RF and anti-CCP2 testing showed no added prognostic value (100). This study highlights the general importance of autoantibodies, given the relatively low progression rate among seronegative arthralgia patients. It also suggests that anti-CCP2 is the most powerful of the three autoantibodies to predict arthritis onset, although it should be borne in mind that 50% of ACPA positive risk arthralgia patients *did not* develop arthritis within 2 years. A recent study from Argentina, which prospectively evaluated patients with hand arthralgia regardless of autoantibody status, similarly found low progression rates among seronegative patients, and considerably increased risk among those positive for RF or ACPA (assay not specified) (101).

Another Dutch cohort comprising 374 arthralgia patients with either anti-CCP2 or RF, were prospectively followed for a median 32 months (102). Clinical arthritis developed in 35% and was better predicted by baseline anti-CCP2 status than by RF, although the highest risk was seen among double positive patients (HR 7.1), suggesting a dose-response relationship. A later study from the same cohort revealed significant prognostic value of anti-CarP also when considering anti-CCP2 and RF (HR 1.6) (103).

In a UK cohort enrolling patients with anti-CCP2 and non-specific musculoskeletal symptoms, 30% progressed to clinical arthritis within 3 years, which was predicted by the concurrent presence of RF or anti-CCP3, respectively (104, 105). Further illustrating the prognostic importance of ACPA, inflammatory arthritis developed in only 1.3% within one year in a large anti-CCP2 negative control population with recent-onset musculoskeletal pain (106).

A Swedish prospective cohort study on anti-CCP2 positive patients with musculoskeletal pain showed 48% progression to clinical arthritis within 6 years (107). Concurrent presence of RF doubled the risk of progression, but anti-CarP did not convey further risk in multivariable analysis. Nevertheless, HRs for arthritis development increased by the number of positive autoantibody classes.

#### Do Antibody Levels Matter?

More prognostic value could potentially be retrieved from autoantibody levels than from status only. It needs to be pointed out, however, that higher levels of autoantibodies often coincide with increased number of autoantibody classes present. The two cohorts studying anti-CCP2 positive patients with musculoskeletal pain found both RF and anti-CCP2 levels to be independently prognostic for arthritis development (105, 107). However, in the study recruiting patients based on symptoms only, regardless of autoantibody status, neither anti-CCP2 nor RF levels turned out to be significant predictors of arthritis (99), although statistical power was limited. Finally, when selecting symptomatic patients positive for either RF or anti-CCP2, only levels of the latter were of prognostic value (102). Taken together, it appears that in settings where symptomatic patients are enriched for seropositivity, anti-CCP2 levels are of importance, and RF levels are important when co-occurring with anti-CCP2.

#### Is There a Value of Repeated Autoantibody Testing in Symptomatic At-Risk Patients?

Retrospective biobank studies on asymptomatic individuals clearly indicated that greater proportions are autoantibody positive (96–98) and autoantibody levels increase (96, 97) as RA diagnosis approaches. Extrapolation of these findings to the symptomatic phase of pre-disease would make it clinically relevant to monitor autoantibody levels to predict arthritis onset. However, growing evidence from prospective studies on symptomatic at-risk patients suggest otherwise. In fact, studies published so far show that RF and ACPA (including non-classical isotypes) appear stable during the symptomatic pre-arthritic phase, both in terms of levels and seroconversion, and without apparent association with arthritis onset (99, 108, 109).

To conclude, anti-CCP2 appears to be the strongest serological predictor for arthritis development among symptomatic at-risk patients. RF confers a clear additive prognostic value, whereas diverging results are found concerning anti-CarP. This, in combination with methodological challenges and absent standardization, preclude broader use of anti-CarP at the present time. Higher baseline anti-CCP2 levels are generally associated with higher arthritis risk and, at least in the anti-CCP2-positive subset, the same holds true for RF. There are at present no indications that repeated autoantibody assessments are informative among symptomatic at-risk patients.

### Autoantibodies in Diagnosis and Prognosis of RA

The diagnostic utility of ACPA in clinical practice is well recognized. For example, in the Swedish National Guidelines for Management of Musculoskeletal Diseases issued by the National Board of Health in 2012 (110), testing for anti-CCP2 antibodies was recommended in all patients with undifferentiated arthritis (i.e. patients with clinical arthritis but not sufficient findings to make a diagnosis of RA or any other established rheumatic disorder). The underlying rational was that those positive for ACPA would be more likely to develop classic RA, and should be followed by a rheumatologist. In the most recent update of these guidelines, approved in January 2021 (111), this point was thought to be well integrated in established clinical practice, and not controversial enough to be included as a central recommendation. Instead, the updated guidelines discussed the evidence for additional value of imaging over and above that of ACPA.

Due to its lower specificity, RF testing in patients with very early arthritis has not been recommended.

By contrast, in patients with persistent inflammatory polyarthritis (i.e. a high pre-test probability of developing classic RA) or in patients with a clinical diagnosis of RA, testing for both ACPA and RF has been recommended (110). This is based on the evidence for a worse prognosis in patients with seropositive RA. In particular, it is well established that both RF and ACPA are strong predictors for rapid progression of joint damage (112). It has been shown that patients with RA who are positive for RF and/or ACPA are more likely to have a gradual increase in radiographic damage scores on a level that has a clinical relevance for long term function and quality of life (113). Furthermore, severe extra-articular manifestations, such as systemic vasculitis or pericarditis, are more likely to occur in seropositive patients (114), and these severe RA phenotypes are particularly linked to high levels of RF (13).

Based on these insights, current recommendations for the management of RA state that RF and ACPA status should be taken into account in treatment decisions (115). For example, among patients who do not have sufficient therapeutic response to methotrexate, which should be the first disease modifying anti-rheumatic drug (DMARD) in most cases, addition of a biologic DMARD (bDMARD) or a targeted synthetic DMARD (tsDMARD) is recommended in those with unfavorable prognostic factors (e.g. RF/ACPA) (115). In accordance with this, most rheumatologists are more willing to escalate therapy rapidly in RF/ACPA positive patients, in particular in those who are positive for both antibodies with high levels. The potential gain from successful treatment compared to natural disease progression is thought to be greater in such patients, creating a more favorable risk-benefit ratio for aggressive anti-rheumatic therapy.

This practice likely contributes to a better prognosis in seropositive patients in recent years, and a reduced difference in the overall disease impact compared to seronegative RA. Studies of inception cohorts of patients with RA in Sweden demonstrated an association between ACPA and disease activity over time among those diagnosed in 1996-1999, but not in those diagnosed in 2006-2009 (116). Furthermore, whereas earlier studies reported a more pronounced general loss of bone mass in seropositive RA (117, 118), more recent inception cohort studies did not demonstrate any difference in change of bone mineral density over time in ACPA positive compared to ACPA negative RA (119).

### ACPA and RF in Prediction of Outcome of Pharmacotherapy

There is also some evidence indicating that ACPA and RF may be useful in the prediction of response to treatment with DMARDs. Such predictive value is particularly relevant for bDMARDs or tsDMARDs, as these are mainly used as second-line agents and are substantially more costly that conventional DMARDs, such as methotrexate. However, the available evidence and the relation between serologic status and treatment outcome is highly variable for different drugs (**Table 1**).

**Table 1:** Summary of evidence for predictive value of ACPA and RF for outcome of treatment with bDMARDs and tsDMARDs in rheumatoid arthritis.

Drug/Class of drugs	Prediction of response	Evidence base	References
TNF inhibitors	No predictive value	SLRs with meta-analyses of observational studies	(120–122)
IL-6 inhibitors	Conflicting evidence; No predictive value or slightly better efficacy in RF/ACPA positive patients	SLR with meta-analysis of RCTs and observational studies (tocilizumab) Observational studies (tocilizumab) Pooled data from RCTs (sarilumab)	(123) (124–127)
Abatacept	Some evidence for modestly better efficacy in ACPA positive patients	SLRs with meta-analysis of observational studies Large observational study of pooled register data	(121, 123) (124)
Rituximab	Better efficacy in RF/ACPA positive patients	RCTs SLR with meta-analysis of RCTs and observational studies Large observational study of pooled register data	(128) (123) (124)
JAK-inhibitors	No predictive value of ACPA (baricitinib) Better efficacy in seropositive as compared to seronegative patients (tofacitinib).	Observational register study (baricitinib) Pooled data from RCTs (tofacitinib).	(129) (130)

bDMARD, biologic disease-modifying anti-rheumatic drug, ACPA, anti-citrullinated protein/peptide antibodies; RF: rheumatoid factor, SLR, systematic literature review; RCT,randomized controlled trial; tsDMARD, targeted synthetic disease-modifying anti-rheumatic drug.

Observational studies indicate that there is no major difference in the efficacy of treatment with tumor necrosis factor inhibitors (TNFi) between patients that are seropositive or seronegative for RF or ACPA (120–122). This is compatible with the well-established efficacy of TNFi overall not only in treatment of RA, but also for seronegative conditions such as psoriatic arthritis (PsA), psoriasis, axial spondyloarthritis (axSpA) and inflammatory bowel disease.

Regarding treatment directed against interleukin-6 (IL-6), using the monoclonal anti-IL-6 receptor antibodies tocilizumab and sarilumab, the data are conflicting. A recently published pooled analysis of data from 16 national registers showed a slightly higher proportion attaining clinical remission among seropositive patients after treatment with tocilizumab (**Table 2**), but seronegativity did not predict discontinuation of tocilizumab (124).

**Table 2:** Adjusted differences in proportions with LUNDEX corrected clinical remission* for patients with seropositive** *vs.* seronegative RA, for different biologic DMARDs.

Drug/Class of drugs	Adjusted*** difference – seropositive *vs* seronegative	95% CI
TNF inhibitor	-0.1%	-0.3, 0.2
Abatacept	1.5%	1.1, 1.9
Tocilizumab	0.9%	0.3, 1.5
Rituximab	5.9%	4.7, 7.3

*Proportions remaining on drug at 1 year, with Clinical Disease Activity Index (CDAI) ≤ 2.8

**RF and/or ACPA positive

***Adjusted for age, sex, smoking (yes/no), BMI for TNF inhibitors, abatacept and tocilizumab (but not for rituximab), for calendar year of treatment start, country, concomitant treatment with csDMARDs and glucocorticosteroids, number of previous bDMARDs and disease characteristics (baseline values for disease activity and disease duration) for all. Pooled analysis from 16 European registers (124).

Most studies suggest that ACPA positive patients with RA are more likely to have a favorable long term outcome of treatment with the CTLA4-based bDMARD abatacept compared to ACPA negative patients (121, 123, 124). In the large observational study of pooled register data, the greatest difference in remission rate for seropositive *vs.* seronegative patients was observed for the B-cell depleting anti-CD20 antibody rituximab (124) (**Table 2**). This is in agreement with previous results from both randomized controlled trials (RCTs) (128) and observational studies (123), although the magnitude of the difference varies. As abatacept blocks T-cell activation, indirectly influencing interaction between T-cells and antibody producing B-cells, and rituximab depletes populations of active B-cells, it is not surprising that these drugs should be somewhat more effective in patients with RA that are seropositive for ACPA or RF.

Data on the tsDMARDs that block the intracellular Janus kinases (JAK), which were introduced more recently than the bDMARDs discussed above, are more limited. Results from the phase III clinical trial program of tofacitinib suggest that they may be slightly more effective in seropositive patients (130). As JAK-inhibition has a wide variety of anti-inflammatory effects, and JAK-inhibitors have been shown to be effective also in the seronegative disorders PsA and axSpA, a minor predictive effect of ACPA and RF would be expected in this context.

## Discussion

We suggest that diagnostic specificities should be harmonized for RF and ACPA tests, and that both groups of assays should be aligned with comparable diagnostic specificities within a defined interval between 98-99% in comparison with healthy controls. The responsibility for establishment of these cut-offs lies with the manufacturing companies, as a large group of healthy controls is needed to establish this high specificity. Such alignment is not in conflict with the current directions for cut-off setting in RA classification.

Complementing these harmonized cutoffs with information about test result-specific likelihood ratios with substantially increase the richness and information value of autoantibody data delivered from the laboratories to the clinicians. It is however conditioned on a change in mindset as clinical physicians have to interpret autoantibody results on ordinal or interval scales. Definition of commensurable likelihood ratios postulates that all compared assays have been compared using the same patient and control populations. Establishment of a serum bank with samples from an international reference population of RA patients and controls for estimation of comparable likelihood ratios would be beneficial in this regard.

Among the RA-related autoantibodies, ACPA has the most pronounced prognostic value concerning RA onset among symptomatic risk patients. And although the risk of RA onset is low in seronegative arthralgia patients, it needs to be stressed that when a patient does present with arthritis, seronegative RA must not be forgotten. Due to lack of evidence in prospective studies, and for cost-benefit reasons, we recommend clinicians to avoid routinely repeated autoantibody measurements in risk populations.

Testing for ACPA is well established in the work-up of early undifferentiated arthritis. In patients diagnosed with RA, both ACPA and RF are associated with increased risk of severe disease progression. Initiation of bDMARDs that directly influence lymphocyte function, in particular rituximab and abatacept, is more likely to result in a major treatment response in ACPA positive patients, whereas no such difference has been observed for TNF inhibitors. Further studies of the relation between autoantibody profiles and treatment outcomes, combined with investigation of other biomarkers and genetics, may contribute to a more personalized approach to the treatment of RA in the future.

## Author Contributions

JR drafted the text about laboratory perspectives, CT drafted the parts about autoantibodies as biomarkers for diagnosis and therapy response, and AK drafted the part about autoantibodies as predictors of arthritis development. All authors contributed to the article and approved the submitted version.

## Funding

This study was funded by the Swedish Rheumatism Association (grant number R-932093, to JR, and grant number R-941284, to CT, and grant number R-931340 to AK), The Swedish Research Council (grant number 2019-01632, to JR, and grant number 2015-02228, to CT) and the King Gustav V 80-year foundation (grant number FAI-2019-0577, to JR, and grant number FAI 2019-0586 to AK).

## Conflict of Interest

JR has been a member of the scientific advisory board for Thermo Fisher Scientific, and has research collaboration with the diagnostic companies Thermo Fischer Scientific, Inova Diagnostics, Euroimmun and Theradiag. CT has received a research grant from Bristol-Myers Squibb, consultancy fees from Roche, and speaker’s honoraria from Abbvie, Bristol-Myers Squibb, Nordic Drugs, Pfizer and Roche. AK has received speaker’s honoraria from Werfen and was previously employed by Sanofi.

The handling editor declared a past co-authorship with one of the authors, JR.
